# Supplementary material for: Molecular determinants of inhibition of UCP1-mediated respiratory uncoupling
Source: Nat Commun. 2023 May 5;14:2594. doi: 10.1038/s41467-023-38219-9 (PMC10162991; doi:10.1038/s41467-023-38219-9)
Supplement: Supplementary file 1 — Supplementary Information [file 41467_2023_38219_MOESM1_ESM.pdf]

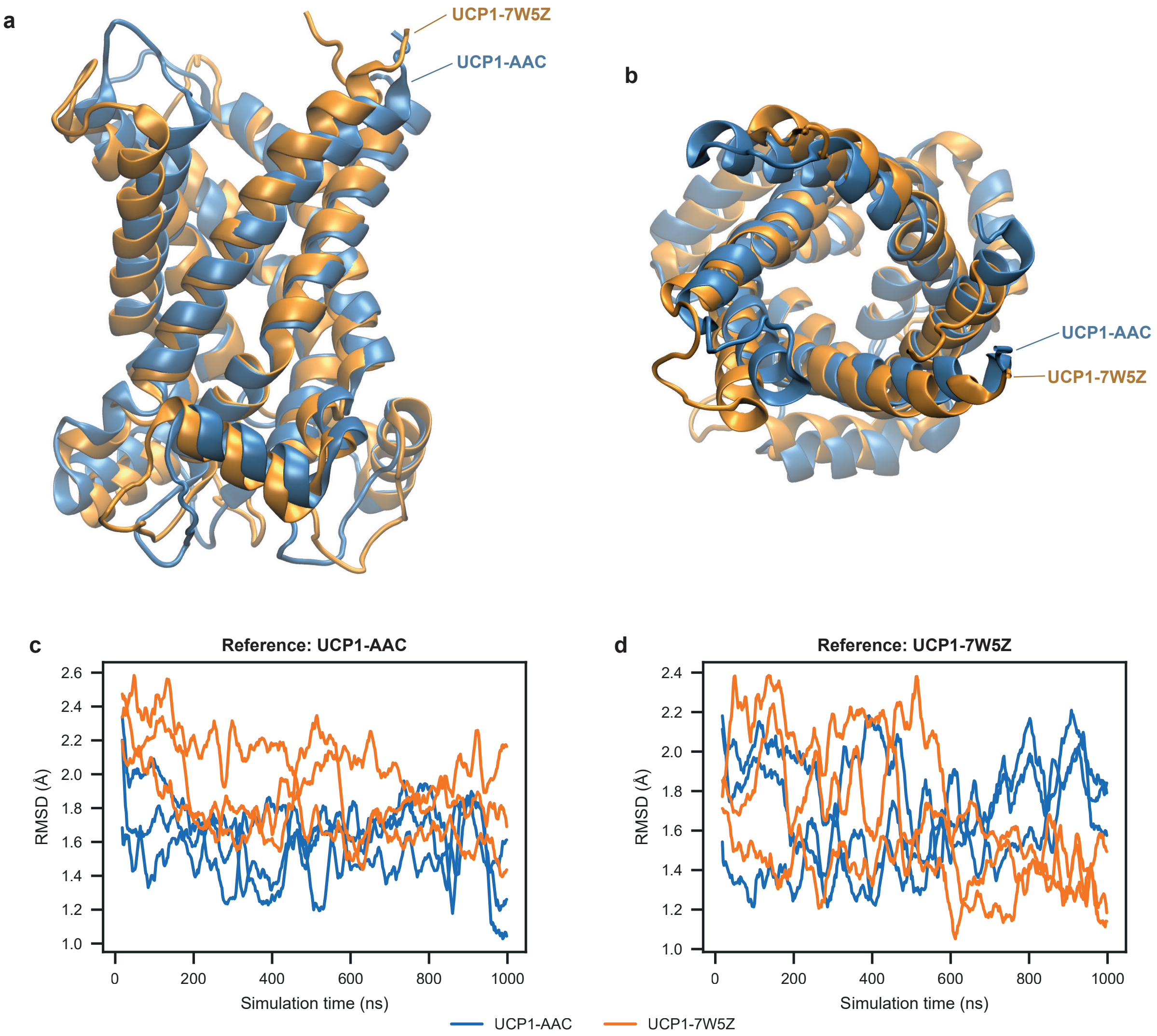

Supplementary Figure 1 | Comparison between models UCP1-AAC C-state, based on the AAC crystal structure (PDB 2C3E), and UCP1-7W5Z C-state, based on a putative 2-oxoglutarate/malate carrier protein from *Tetrahymena thermophila* (PDB 7W5Z). a, **b** Average structures of the three replicas of UCP1-AAC and UCP1-7W5Z after 1 µs of MD simulation. **a** View from the membrane. **b** View from the cytosol. **c**, **d** Time trajectories of the cross-RMSD of residues of the UCP1 cavity (triplets 38, 84, 85, 88 and 89) in simulations of the UCP1-AAC and UCP1-7W5Z models, with respect to replica-averaged relaxed configurations (after 1 µs) of those two models. The curves were smoothed with a running average (20-ns window). Source data are provided as a Source Data file.

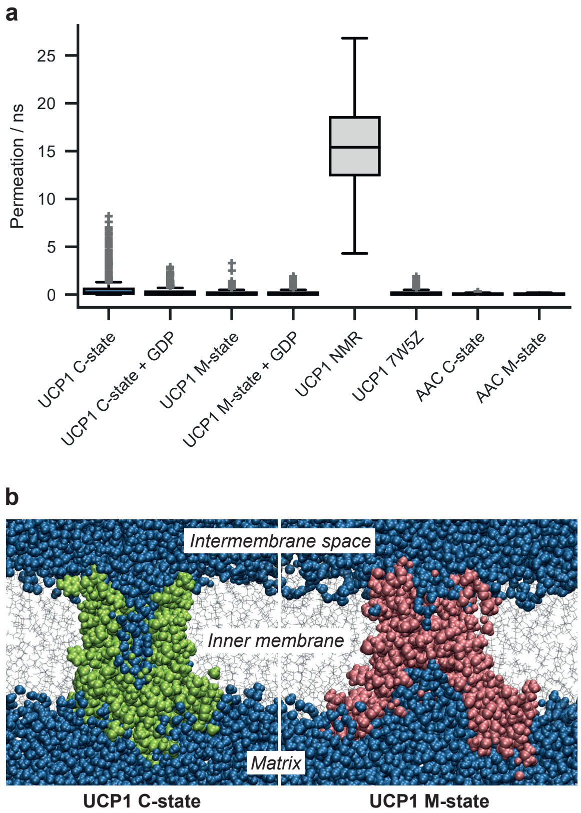

Supplementary Figure 2 | Water permeability measurements in MD simulations. a Number of water molecules crossing the membrane through the protein per nanosecond. UCP1 NMR is an homology model of UCP1 built from NMR structure of UCP2 (PDB 2LCK). Boxes of the boxplots indicates the first quartile, the median and the third quartile. The whisker length is 1.5 times the interquartile range. Number of replicas, n = 1 for UCP1 NRM, AAC C-state and AAC M-state; n = 3 for UCP1-AAC-M-state, UCP1-AAC-C-state, UCP1-AAC-M-state + GDP and UCP1-7W5Z C-state; n = 4 for UCP1-AAC C-state + GDP except for UCP1-Alphafold n = 1 **b** Cross-section view of UCP1 hydration. Water molecules are depicted as blue spheres and atoms of UCP1 C-state and UCP1 M-state are represented as, respectively, green and red spheres. Source data are provided as a Source Data file.

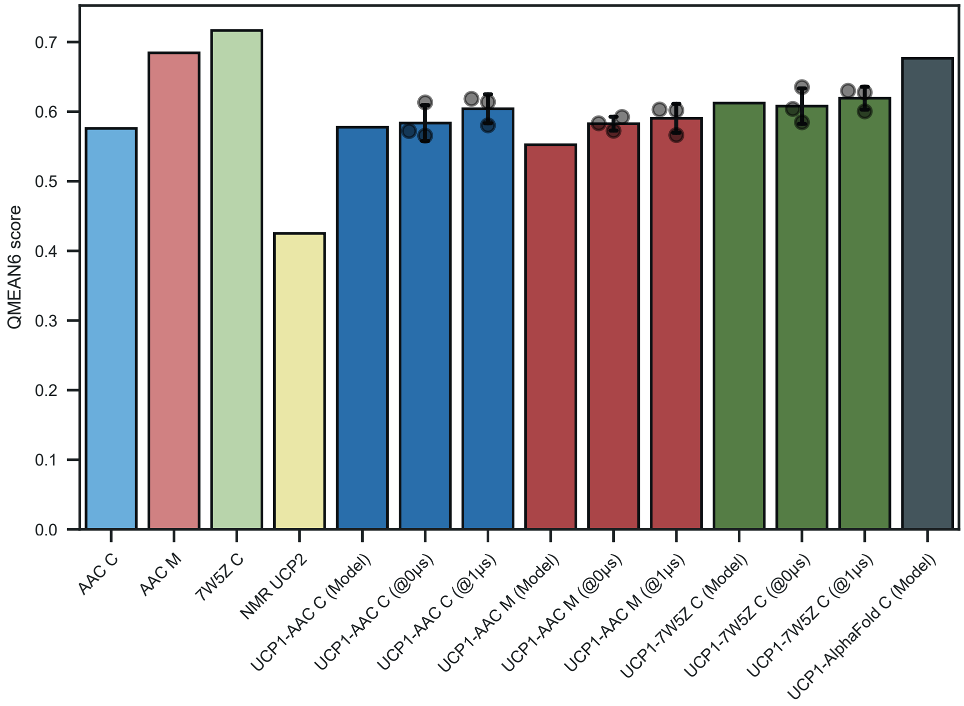

Supplementary Figure 3 | QMEAN6 normalized scores of mitochondrial carrier experimental structures and UCP1 models before and after MD simulations. *AAC C-state and AAC M-state*: AAC C-state and M-state crystal structures (PDB 2C3E and 6GCI). *7W5Z C-state*: uncharacterized C-state-like mitochondrial carrier from Tetrahymena thermophila (PDB 7W5Z). *NMR UCP2*: NMR structure of UCP2 (PDB 2LCK). *UCP1-AAC C-state and UCP1-AAC M-state*: UCP1 models based on AAC C-state and AAC M-state crystal structures (PDB 2C3E and 6GCI). *UCP1-7W5Z*: UCP1 model based on uncharacterized C-state-like mitochondrial carrier from Tetrahymena thermophila (PDB 7W5Z). *UCP1-AlphaFold*: UCP1 model generated by AlphaFold 2. "Model", "@0μs" and "@1μs" indicates the score of, respectively, the model before MD simulations, the structures after equilibration and and the structures after 1 μs of MD simulation. Number of replicas, n = 3. The bars indicate the QMEAN6 values for single structures and the mean ± SD between replicas for the structures from MD simulations. Individual contact time of each replica is represented by a black dot. Source data are provided as a Source Data file.

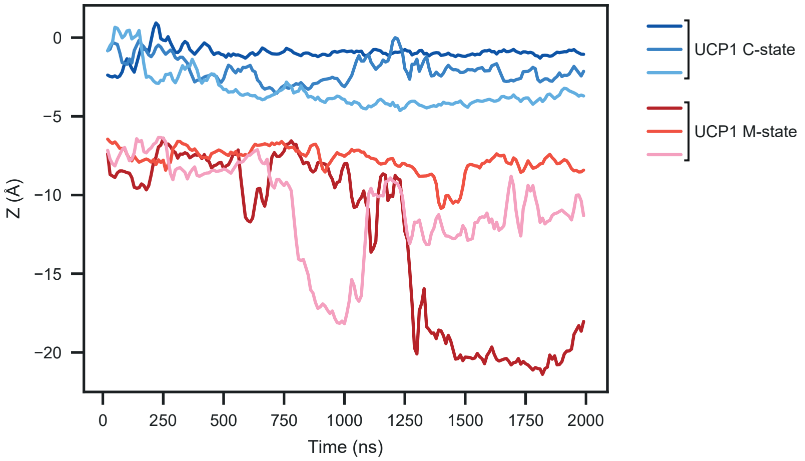

Supplementary Figure 4 | Vertical distance of GDP center from the protein center as a function of simulation time during restraint-free simulations of UCP1 C- and M-state. Structures were aligned on the alpha carbons of the transmembrane helices of UCP1. The curves were smoothed with a running average (30-ns window).

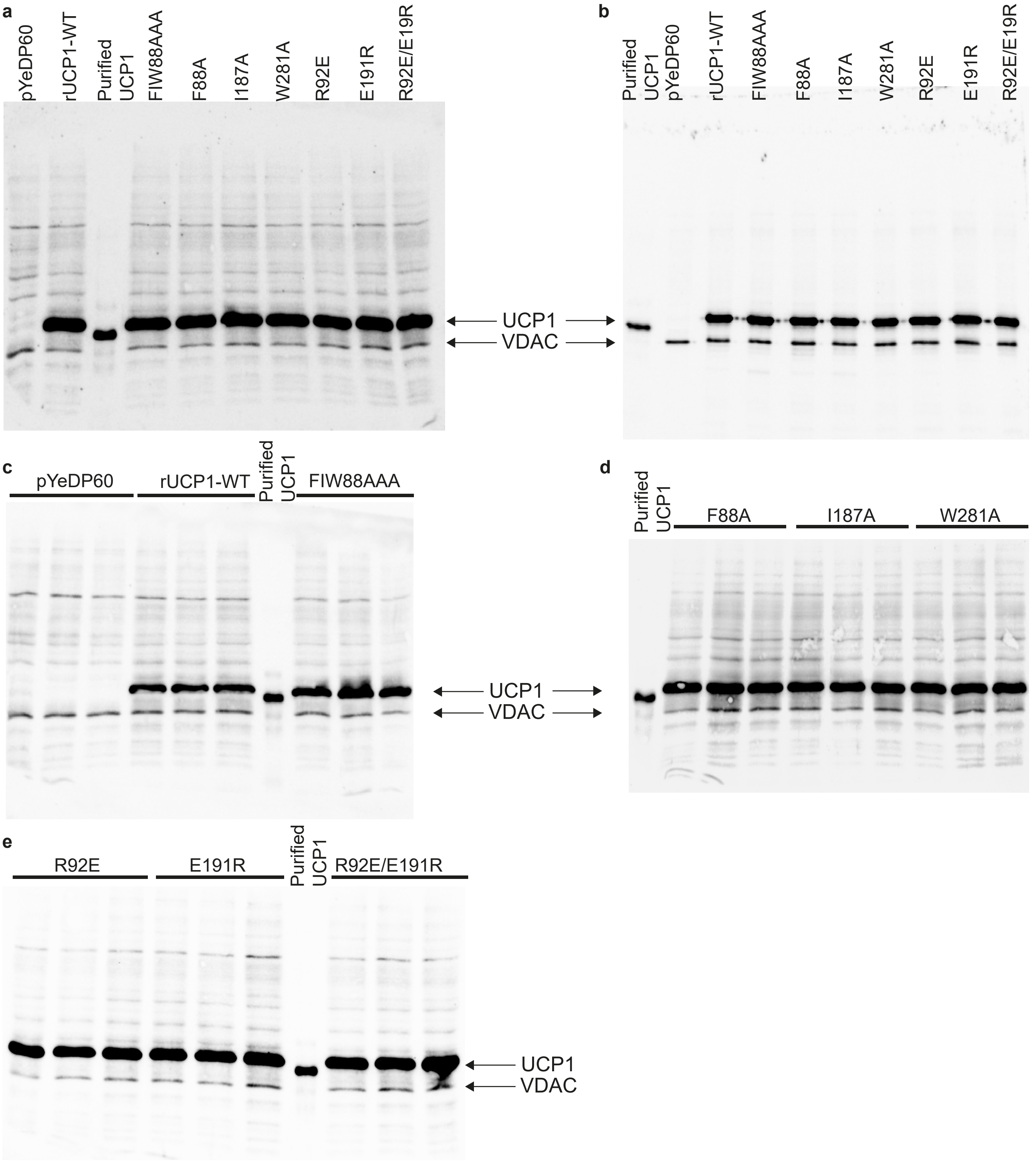

Supplementary Figure 5 | Immunodetection of UCP1 on recombinant yeast grown in S-lactate medium and prepared according to Methods. Immunodetection of porine VDAC is used as a loading control. Both proteins are revealed using a mouse anti-pentahistine tag:HRP and a mouse anti-VDAC1, see Methods. Expression is measured on total TCA extracts **a** and on mitochondria **b**. **c**, **d**, **e** are replicates of total TCA extracts. Purified UCP1 is used as weight marker as it weights 33.3 kDa (1.7 kDa lighter than expressed UCP1). n = 6, except for pYeDP60 where n = 3, biologically independent experiments. Samples were prepared identically and were run in paralell to allow quantitative comparisons. Statistical analyses are presented in Supplementary Table 1. Source data are provided as a Source Data file.

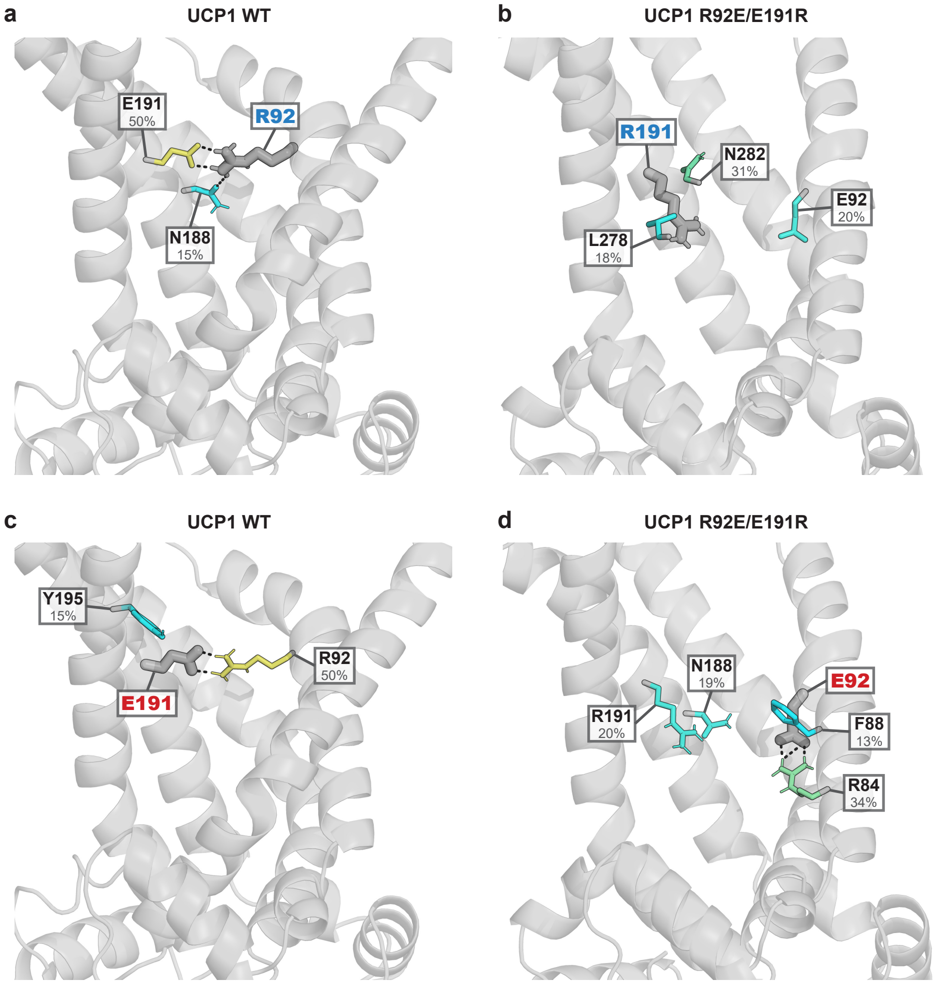

Supplementary Figure 6 | Intra-protein contacts in simulations of UCP1 wild type and the double mutant R92E/E191R. **a**, **b**, **c** and **d** Contact time between protein residues and the charged atoms of, respectively, R92, R191, E191 and E92. Snapshots of trajectories at 500 ns of UCP1 wild type for **a** and **c**, and of UCP1 R92E-E191R for **b** and **d**. Only the residues with a contact time higher than 10% are represented. Residues are colored according to their contact time. The color scale goes from cyan at 10% to yellow at 55% to red at 100%. Contact times are computed on simulations from 200 ns to 1000 ns.

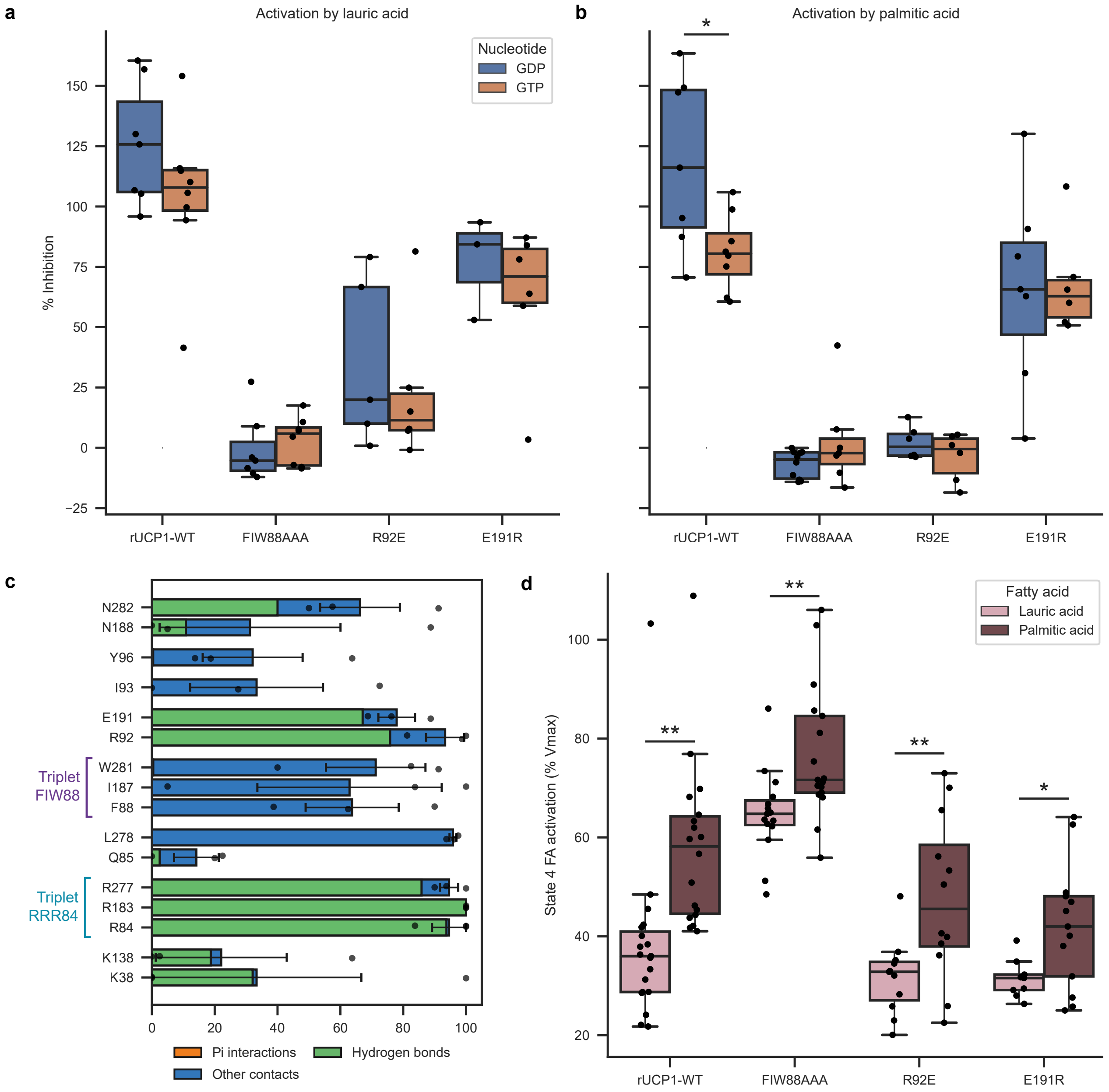

Supplementary Figure 7 | Effect of fatty acid chain length and number of nucleotide phosphates on UCP1 activation and inhibition. UCP1 inhibition by GDP (blue) or GTP (orange) after activation either by lauric acid **a** or palmitic acid **b**. **c** Contact time between GTP and protein residues as a fraction of simulation time, excluding the first 200 ns. The part of that contact time involving hydrogen bonds is represented in green, and the part involving pi interactions is represented in orange. Contact times are averaged over the trajectories. Number of replicas, n = 3. Bars indicated the mean ± SEM of total contact times. Individual contact time of each replica is represented by a transparent black dot. **d** UCP1 activation in response to either lauric acid or palmitic acid. **a** rUCP1-WT n = 15; FIW88AAA n = 15; R92E n = 11; E191R n = 9 over four biologically independent experiments. **b** rUCP1-WT n = 15; FIW88AAA n = 17; R92E n = 12; E191R n = 13 over four biologically independent experiments. **d** rUCP1-WT n = 19 n = 18; FIW88AAA n = 15 n = 17; R92E n = 11 n = 12; E191R n = 9 n = 13 over four biologically independent experiments. LA = Lauric acid; PA = Palmitic acid Boxes of the boxplots indicates the first quartile, the median and the third quartile. The whisker length is 1.5 times the interquartile range. Individual data points are superimposed over each boxplot. Data were analyzed with a two-sided t test. ns = not significant, $*$ = p-value $\leq$ 0.05, $**$ = p $\leq$ 0.01, $***$ = p-value $\leq$ 0.001, $****$ = p-value $\leq$ 0.0001. Statistical analyses are presented in Supplementary Tables 8 and 9. Source data are provided as a Source Data file.

Supplementary Figure 8 | Multiple alignments of human mitochondrial carrier sequences and rat UCP1. Gray bars are indicate hidden parts of sequences. Colored backgrounds are an estimation of alpha helices from the UCP1 C-state model gray and yellow backgrounds are respectively odd and even transmembrane helices, and green are non-transmembrane helices. Dark gray vertical bars depict hidden parts of the sequences. Abbreviations: *rUCP1* rat Uncoupling Protein 1; *hsUCP1* Uncoupling Protein 1; *hsUCP2* Uncoupling Protein 2; *hsUCP3* Uncoupling Protein 3; *hsUCP4* Uncoupling Protein 4; *hsUCP5* Uncoupling Protein 5; *hsDIC* Mitochondrial dicarboxylate carrier; *hsODC* Mitochondrial 2-oxodicarboxylate carrier; *hsTXTP* Tricarboxylate transport protein; *hsAAC1* ADP/ATP Carrier 1; *hsGDC* Graves disease carrier; *hsSCMC1* Mitochondrial ATP-Mg/Pi carrier protein 1; *hsS2536* Solute carrier family 25 member 36; *hsMFTC* Mitochondrial folate transporter/carrier; *hsS2540* Solute carrier family 25 member 40; *hsS2538* Mitochondrial glycine transporter; *hsCMC1* Mitochondrial aspartate glutamate carrier 1; *hsMPCP* Phosphate carrier protein.

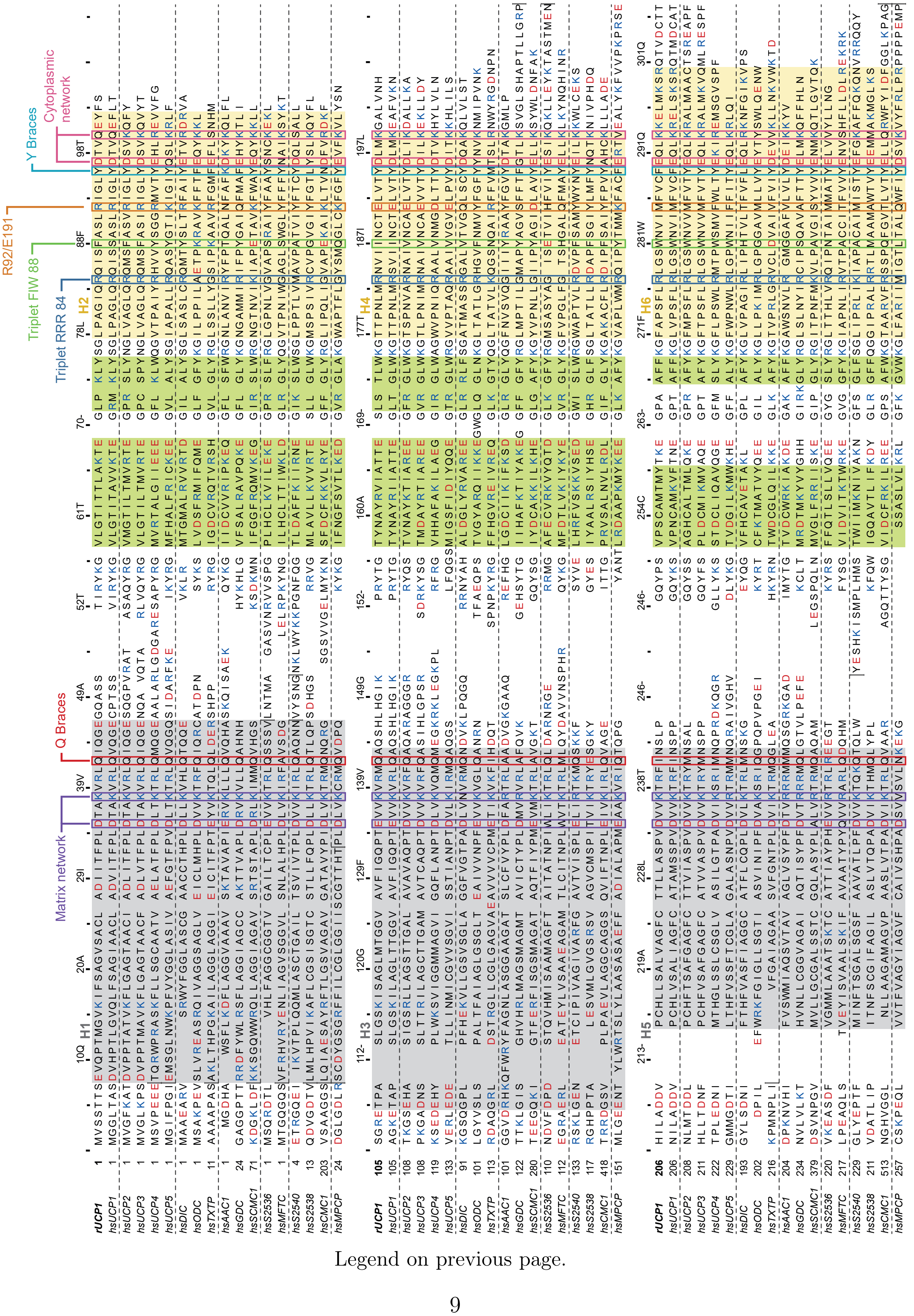

Legend on previous page.

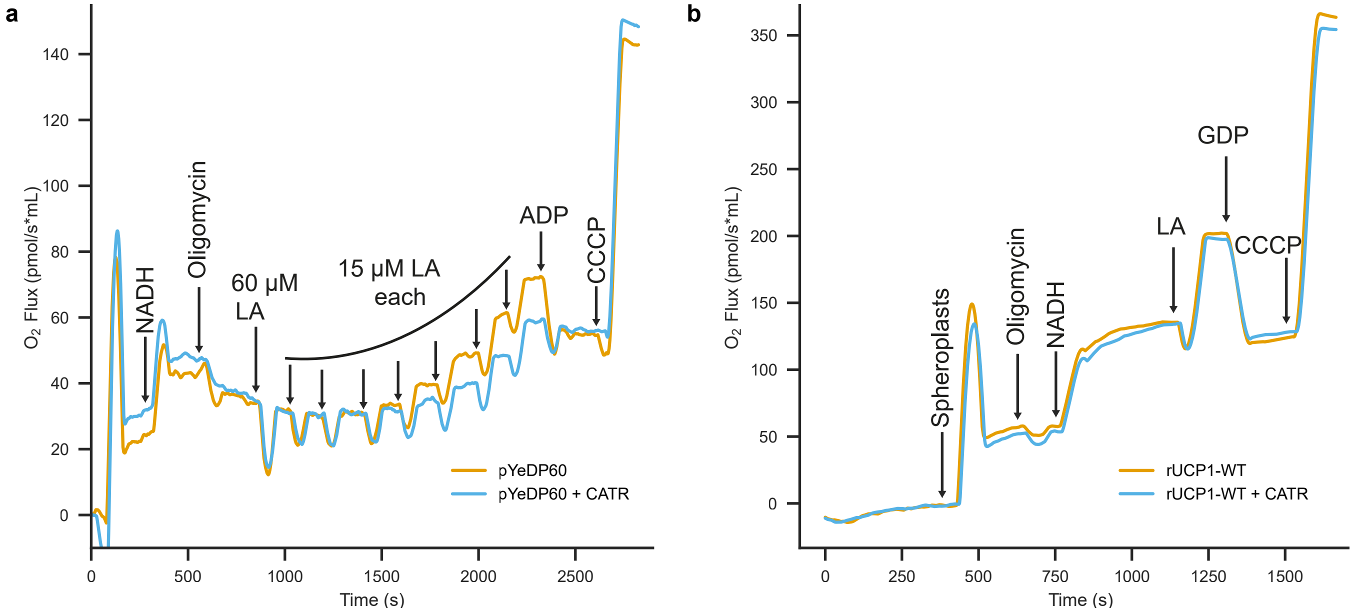

Supplementary Figure 9 | AAC-dependent respiratory uncoupling is inhibited by ADP and requires a higher FFA concentration than UCP1-dependent uncoupling. **a** Respiration curves showing the effect of 60 micromolar concentration of LA and of subsequent multiple additions of 15 micromolar amount of FFA to yeast control spheroplasts in the presence (blue curve) or absence (orange curve) of the CATR inhibitor of AAC. As shown in [15], the addition of ADP suppresses, as well as CATR, the LA-induced increase of respiration. **b** Respiration curves of rUCP1-WT spheroplasts in the presence (blue curve) or absence (orange curve) of CATR. In this experimental setup, the addition of 60 micromolar concentration of LA (LA/BSA=4) had no effect on yeast AAC. CCCP = Carbonyl Cyanide m-Chlorophenyl hydrazone.

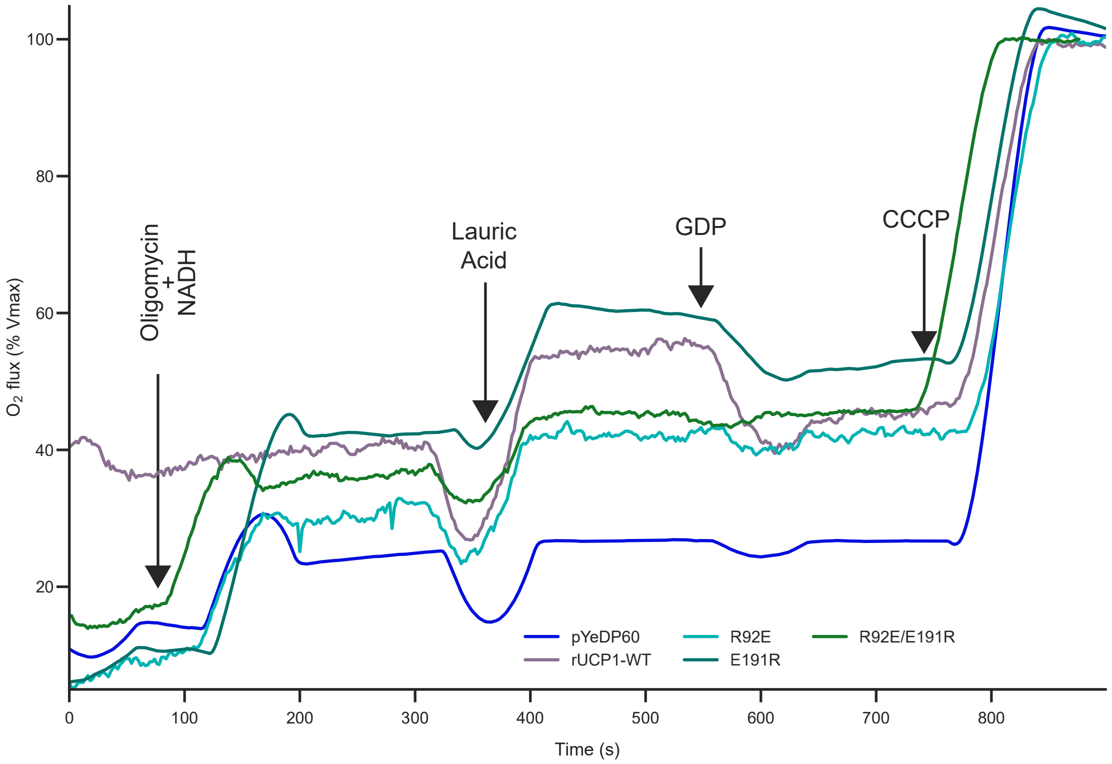

Supplementary Figure 10 | Representative oxygen flux consumption curves of permeabilized spheroplasts harboring either control pYeDP60 plasmid expression (pink curve) or expressing rUCP1-WT (grey curve), mutants R92E (red curve), E191R (blue curve), R92E/E191R (purple curve).

Mutant	Mean	SEM	F	N	Df	p-value	95 % Confidence interval
rUCP1-WT	1.859	0.1204	1.397	6	40	-	-
R92E	3.12	0.8387		6		0.1221	-0.2176 to 2.738
E191R	2.291	0.431		6		0.9447	-1.046 to 1.91
R92E/E191R	2.049	0.2935		6		0.9995	-1.289 to 1.667
FIW88AAA	2.221	0.2987		6		0.977	-0.2176 to 2.738
F88A	1.838	0.21		6		>0.9999	-1.499 to 1.457
I187A	1.725	0.1447		6		0.9996	-1.612 to 1.344
W281A	1.829	0.1656		6		>0.9999	-1.508 to 1.448

Supplementary Table 1 | **Statistical analyses of UCP1 wild-type and mutant expression in yeast total TCA extracts.** UCP1/VDAC ratios are calculated using the SDS-PAGES shown in Figure 5. ImageJ 1.53k and statistical analyses calculate intensity, are performed on PRISM with One-way ANOVA and Dunnett's multiple comparison test with rUCP1-WT. Source data are provided as a Source Data file.

Mutant	Mean	SEM	F	N	Df	p-value	95 % Confidence interval
pYeDP60	2.516	0.2592	4.718	6	24	0.0036	-1.485 to -0.2377
rUCP1-WT	1.657	0.1148		6		-	-
R92E	1.659	0.1019		3		>0.9999	-0.7655 to 0.7625
E191R	1.451	0.07979		3		0.9752	-0.5578 to 0.9703
R92E/E191R	1.558	0.1105		3		0.9995	-0.6649 to 0.8631
FIW88AAA	1.371	0.128		3		0.8732	-0.4777 to 1.05
F88A	1.746	0.2481		3		0.9996	-0.853 to 0.6751
I187A	1.335	0.1116		3		0.9752	-0.442 to 1.086
W281A	1.453	0.1681		3		0.995	-0.5599 to 0.9681

Supplementary Table 2 | **Statistical analyses of spheroplasts respiratory control ratio (RCR) with One-way ANOVA and Dunnett's multiple comparison test versus rUCP1-WT.** Source data are provided as a Source Data file.

Mutant	Mean	SEM	F	N	Df	p-value	95 % Confidence interval
pYeDP60	1.237	0.5629	13.22	5	42	<0.0001	16.77 to 35.28
rUCP1-WT	27.26	2.265		8		-	-
R92E	19.37	1.776		11		0.0381	0.3448 to 15.43
E191R	20.77	2.563		11		0.1091	-1.058 to 14.03
R92E/E191R	17.48	1.672		12		0.0064	2.368 to 17.19

Supplementary Table 3 | **Statistical analyses of spheroplasts respiration activation by lauric acid with One-way ANOVA and Dunnett's multiple comparison test: rUCP1-WT versus salt bridge mutants.**

Mutant	Mean	SEM	F	N	Df	p-value	95 % Confidence interval
rUCP1-WT	98.69	7.736	38.86	8	38	-	-
R92E	-16.15	4.932		11		<0.0001	85.54 to 144.1
E191R	32.96	11.20		11		<0.0001	36.42 to 95.03
R92E/E191R	-12.98	6.662		12		<0.0001	82.88 to 140.4

Supplementary Table 4 | **Statistical analyses of UCP1 inhibition by GDP with One-way ANOVA and Dunnett's multiple comparison test: rUCP1-WT versus salt bridge mutants.**

Mutant	Mean	SEM	F	N	Df	p-value	95 % Confidence interval
pYeDP60	4.738	0.7848	59.36	10	63	<0.0001	29.69 to 57.05
rUCP1-WT	48.11	2.542		12		-	-
FIW88AAA	82.48	4.289		12		<0.0001	-47.41 to -21.33
F88A	45.77	4.268		12		0.9882	-10.71 to 15.38
I187A	76.71	4.351		12		<0.0001	-41.65 to -15.56
W281A	74.05	3.567		11		<0.0001	-39.27 to -12.6

Supplementary Table 5 | **Statistical analyses of spheroplasts respiration activation by lauric acid with One-way ANOVA and Dunnett's multiple comparison test: rUCP1-WT versus 88 mutants.**

Mutant	Mean	SEM	F	N	Df	p-value	95 % Confidence interval
rUCP1-WT	89.15	3.343	108.2	12	54	-	-
FIW88AAA	-4.911	1.881		12		<0.0001	78.5 to 109.6
F88A	97.87	7.237		12		0.4325	-24.28 to 6.834
I187A	24.05	4.226		12		<0.0001	49.54 to 80.65
W281A	19.58	3.245		11		<0.0001	63.66 to 85.47

Supplementary Table 6 | **Statistical analyses of UCP1 inhibition by GDP with One-way ANOVA and Dunnett's multiple comparison test: rUCP1-WT versus 88 mutants.**

Addition	Mutant	Mean	SEM	F	N	Df	p-value	95 % Confidence interval
GDP	pYeDP60	0.2631	0.2178	47.62	6	25	-	-
	rUCP1-WT	-18.29	2.371		11		<0.0001	12.41 to 24.7
	FIW88AAA	1.989	0.7494		11		0.7199	-7.868 to 4.417
LA1	pYeDP60	2.089	0.2738	147.2	6	25	-	-
	rUCP1-WT	-11.03	3.124		11		0.0250	1.591 to 24.65
	FIW88AAA	57.89	3.470		11		<0.0001	-67.33 to -44.27
LA2	pYeDP60	8.572	0.3917	132.2	6	25	-	-
	rUCP1-WT	12	3.326		11		0.7873	-17.88 to 11.02
	FIW88AAA	88.04	4.817		11		<0.0001	-93.92 to -65.02

Supplementary Table 7 | **Statistical analyses of GDP addition then lauric acid on UCP1.** Analyses are done with One-way ANOVA and Dunnett's multiple comparison test with pYeDP60.

FA	Mutants	Nucleotide	Mean	SEM	F	N	t	Df	p-value	95 % Confidence interval
LA	rUCP1-WT	GDP	125.9	9.593	1.515	7	1.439	13	0.1737	-10.7 to 53.44
		GTP	104.5	11.04		8				
	FIW88AAA	GDP	-0.5855	5.344	2.098	7	0.5785	13	0.5728	-16.98 to 9.81
		GTP	3.002	3.451		8				
	R92E	GDP	35.3	15.75	1.37	5	0.6476	9	0.5334	-31.74 to 57.21
		GTP	22.57	12.28		6				
	E191R	GDP	76.91	12.26	2.137	3	0.7113	7	0.4999	-33.41 to 62.15
		GTP	62.54	12.67		6				
PA	rUCP1-WT	GDP	118.5	13.46	5.025	7	2.687	13	0.0187	-67.36 to -7.313
		GTP	81.15	5.616		8				
	FIW88AAA	GDP	-6.806	5.712	11.25	10	1.471		0.1621	-4.201 to 22.90
		GTP	1.806	7.242		7				
	R92E	GDP	2.136	2.726	2.205	6	1.215	10	0.2524	-16.8 to 4.947
		GTP	-3.792	4.048		6				
	E191R	GDP	66.2	67.93	3.703	7	0.09281	11	0.9277	-39.14 to 42.59
		GTP	15.43	8.661		6				

Supplementary Table 8 | **Statistical analyses of inhibition by GDP or GTP after activation either by lauric or palmitic acid.** Analyses are done with a t test between GDP and GTP for each mutant.

Mutant	Fatty acid	Mean	SEM	F	t	N	Df	p-value	95 % Confidence interval
rUCP1-WT	LA	38.13	4.014	1.078	3.529	19	35	0.0012	8.475 to 31.43
	PA	58.08	3.972			18			
FIW88AAA	LA	64.54	2.263	2.382	2.919	15	30	0.0066	3.587 to 20.3
	PA	76.78	3.282			17			
R92E	LA	31.79	2.287	4.746	2.915	11	21	0.0083	4.553 to 27.22
	PA	47.68	4.771			12			
E191R	LA	31.39	1.286	10.7	2.432	9	20	0.0245	1.513 to 19.74
	PA	42.02	3.501			13			

Supplementary Table 9 | **Statistical analyses of spheroplasts respiration activation by lauric or palmitic acid.** Analyses are done with a t test between AL and AP for each mutant.
