# Supplementary material for: Molecular determinants of inhibition of UCP1-mediated respiratory uncoupling
Source: Nat Commun. 2023 May 5;14:2594. doi: 10.1038/s41467-023-38219-9 (PMC10162991; doi:10.1038/s41467-023-38219-9)
Supplement: Supplementary file 3 — Description of Additional Supplementary Files [file 41467_2023_38219_MOESM3_ESM.pdf]

Description of Additional Supplementary Files:

Supplementary Movie 1 | GDP entry into the UCP1 common substrate binding site, as observed in an ABMD simulation (simulated time shown: 20~ns), and rendered using VMD. The R92/E191 salt bridge and arginine triplet R84 are represented in licorice, colored in orange and cyan respectively. GDP is colored by element.
