# Supplementary material for: Molecular determinants of inhibition of UCP1-mediated respiratory uncoupling
Source: Nat Commun. 2023 May 5;14:2594. doi: 10.1038/s41467-023-38219-9 (PMC10162991; doi:10.1038/s41467-023-38219-9)
Supplement: Supplementary file 5 — Reporting Summary [file 41467_2023_38219_MOESM5_ESM.pdf]

nature portfolio

Corresponding author(s): Jérôme Hénin and Bruno Miroux

Last updated by author(s): Mar 31, 2023

Reporting Summary

Nature Portfolio wishes to improve the reproducibility of the work that we publish. This form provides structure for consistency and transparency in reporting. For further information on Nature Portfolio policies, see our Editorial Policies and the Editorial Policy Checklist.

Statistics

For all statistical analyses, confirm that the following items are present in the figure legend, table legend, main text, or Methods section.

n/a	Confirmed	
☐	☒	The exact sample size (*n*) for each experimental group/condition, given as a discrete number and unit of measurement
☐	☒	A statement on whether measurements were taken from distinct samples or whether the same sample was measured repeatedly
☐	☒	The statistical test(s) used AND whether they are one- or two-sided<br>*Only common tests should be described solely by name; describe more complex techniques in the Methods section.*
☒	☐	A description of all covariates tested
☐	☒	A description of any assumptions or corrections, such as tests of normality and adjustment for multiple comparisons
☐	☒	A full description of the statistical parameters including central tendency (e.g. means) or other basic estimates (e.g. regression coefficient) AND variation (e.g. standard deviation) or associated estimates of uncertainty (e.g. confidence intervals)
☐	☒	For null hypothesis testing, the test statistic (e.g. *F*, *t*, *r*) with confidence intervals, effect sizes, degrees of freedom and *P* value noted<br>*Give P values as exact values whenever suitable.*
☒	☐	For Bayesian analysis, information on the choice of priors and Markov chain Monte Carlo settings
☒	☐	For hierarchical and complex designs, identification of the appropriate level for tests and full reporting of outcomes
☒	☐	Estimates of effect sizes (e.g. Cohen's *d*, Pearson's *r*), indicating how they were calculated

Our web collection on statistics for biologists contains articles on many of the points above.

Software and code

Policy information about availability of computer code

Data collection	The software NAMD 2.14 and the Colvars library were used to produce molecular dynamics simulations.
Data analysis	Analysis of the MD simulations was performed with VMD 1.9.4a43 and the python library MDAnalysis 2.3.0. Charts were produced with the python libraries Matplotlib 3.6.2 and Seaborn 0.12.1. Alignments were produced with Jalview 2.11.2.6 and the web service MAFFT 7. Homology models were produced with Modeller 9.22 and 10.2.

For manuscripts utilizing custom algorithms or software that are central to the research but not yet described in published literature, software must be made available to editors and reviewers. We strongly encourage code deposition in a community repository (e.g. GitHub). See the Nature Portfolio guidelines for submitting code & software for further information.

Data

Policy information about availability of data

All manuscripts must include a data availability statement. This statement should provide the following information, where applicable:

- Accession codes, unique identifiers, or web links for publicly available datasets
- A description of any restrictions on data availability
- For clinical datasets or third party data, please ensure that the statement adheres to our policy

All data necessary for reproducing simulation results are available through Zenodo (DOI: 10.5281/zenodo.7698270)
Accession codes :

2C3E [http://doi.org/10.2210/pdb2C3E/pdb] (Bos taurus mitochondrial ADP/ATP carrier)
2LCK [http://doi.org/10.2210/pdb2LCK/pdb] (Mus musculus mitochondrial UCP2)
4C9Q [http://doi.org/10.2210/pdb4C9Q/pdb] (Saccharomyces cerevisiae ADP/ATP carrier isoform 3 inhibited by carboxyatractyloside)
6GCI [http://doi.org/10.2210/pdb6GCI/pdb] (Thermothelomyces thermophilus mitochondrial ADP/ATP carrier)
7W5Z [http://doi.org/10.2210/pdb7W5Z/pdb] (Tetrahymena thermophila mitochondrial complex IV) chain M2 (Uniprot entry Q23M99)

Human research participants

Policy information about studies involving human research participants and Sex and Gender in Research.

Reporting on sex and gender	N/A
Population characteristics	N/A
Recruitment	N/A
Ethics oversight	N/A

Note that full information on the approval of the study protocol must also be provided in the manuscript.

Field-specific reporting

Please select the one below that is the best fit for your research. If you are not sure, read the appropriate sections before making your selection.

☒ Life sciences ☐ Behavioural & social sciences ☐ Ecological, evolutionary & environmental sciences

For a reference copy of the document with all sections, see nature.com/documents/nr-reporting-summary-flat.pdf

Life sciences study design

All studies must disclose on these points even when the disclosure is negative.

Sample size	As a starting point, we perform respiration experiments with three independent biological samples and technical triplicates for each sample. Statistical analyses showed significant differences between conditions, confirming that the sample sizes were sufficient.
Data exclusions	Respiration VO2max was used as exclusion criterion. On rare occasions (10 out of 400 measurements), due to a default in spheroplasts preparation, low VO2 max was recorded and these datasets were excluded.
Replication	All experiments were replicated. All attempts at replication were successful except when spheroplasts permeabilization failed (see data exclusions).
Randomization	Yeast culture are clonal for one expression plasmid encoding UCP1 or UCP1-mutant. Therefore, there were no distinct populations to randomize.
Blinding	Blinding was not relevant for this study. Respiration data were generated by the Oroboros instrument and analysed by the same algorithm for all conditions. The Oroboros instrument was run independently by three different users.

Reporting for specific materials, systems and methods

We require information from authors about some types of materials, experimental systems and methods used in many studies. Here, indicate whether each material, system or method listed is relevant to your study. If you are not sure if a list item applies to your research, read the appropriate section before selecting a response.

Materials & experimental systems

n/a	Involved in the study
☐	☒ Antibodies
☐	☒ Eukaryotic cell lines
☒	☐ Palaeontology and archaeology
☒	☐ Animals and other organisms
☒	☐ Clinical data
☒	☐ Dual use research of concern

Methods

n/a	Involved in the study
☒	☐ ChIP-seq
☒	☐ Flow cytometry
☒	☐ MRI-based neuroimaging

Antibodies

Antibodies used

Mouse anti-pentahistidine tag:HRP (Bio-RAD, catalogue number: MCA5995P, clone: ABD 2.2.20)
Mouse anti-VDAC-1 (Invitrogen, catalogue number: 459500, clone: 16G9E6BC4)
Goat anti-mouse tag:HRP (Promega, catalogue number: W4021, polyclonal)

Validation

The anti-pentahistidine antibody, as well as VDAC-1 antibody was validated by the manufacturer (see: https://www.bio-rad-antibodies.com/monoclonal/synthetic-peptide-histidine-tag-antibody-abd2-2-20-mca5995.html and https://www.thermofisher.com/antibody/product/Porin-Antibody-clone-16G9E6BC4-Monoclonal/459500)

Eukaryotic cell lines

Policy information about cell lines and Sex and Gender in Research

Cell line source(s)

W303.1b yeast (Saccharomyces cerevisiae) (alpha, leu2, his3, trp1::TRP1-GAL10-GAL4, ura3, ade2-1, canR, cir+)

Authentication

Auxotroph to uracile and adenine

Mycoplasma contamination

N/A

Commonly misidentified lines
(See ICLAC register)

N/A
